# A comparison of overall survival from early-onset and late-onset colorectal cancer stratified by TNM stage: a systematic review

**DOI:** 10.1038/s41416-026-03426-w

**Published:** 2026-05-04

**Authors:** Nadeem Al-Khafaji, Frances Steele, Haoxuan Li, Hannah Boyden, Umar Bin Tariq, Joanna Hooper, Adam C. Chambers, David E. Messenger

**Affiliations:** 1 Department of Colorectal surgery, University Hospitals Plymouth NHS trust, Plymouth, UK; 2 Department of Coloproctology, University Hospitals Bristol and Weston NHS Foundation trust, Bristol, UK; 3 King’s College Hospital NHS Foundation Trust, Denmark Hill, London, UK; 4 Institute of Psychiatry, Psychology and Neuroscience, King’s College London, London, UK; 5 Craigavon Area Hospital, Southern Health and Social Care Trust, Northern Ireland, Portadown, UK; 6 Library and Knowledge Service, Learning and Research, Southmead Hospital, Bristol, UK; 7 School of Cellular and Molecular Medicine, University of Bristol, Bristol, UK

**Keywords:** Colorectal cancer, Surgical oncology

## Abstract

**Background:**

There is conflicting evidence whether survival outcomes are better for early-onset colorectal cancer (EOCRC) or late-onset (LO) CRC. This review aimed to determine overall survival (OS) differences between EOCRC and LOCRC stratified by TNM stage.

**Methods:**

EMBASE, Ovid MEDLINE and Cochrane Library were searched using approved search terms for CRC, survival outcomes and early onset disease. Studies which compared OS between early- and late-onset CRC, irrespective of age cut-off, stratified by TNM stage, were included. The Methodological Index for Non-randomized Studies was used to assess methodology and risk of bias. Data on 5-year OS, TNM stage, histopathological features, cohort study type and use of confounder-adjusted analyses were extracted and descriptive statistics presented as study heterogeneity prevented meta-analysis.

**Results:**

A total of 21 studies described the OS of 332451 patients: 29199 EOCRC and 303252 LOCRC. Studies mostly found favourable outcomes in EOCRC, but where multiple age groups were compared, worse survival was frequently observed in the very youngest patients, especially in stage II and III disease.

**Conclusion:**

EOCRC may have worse OS in age and stage subgroups. This warrants further study using large, granular datasets with detailed information on staging and histopathological features. PROSPERO ID CRD42024563472. An earlier version of this review was presented at the Association of Coloproctologists of Great Britain and Ireland Annual General Meeting in July 2023 and the Tripartite Colorectal Meeting 2025.

## Introduction

Colorectal cancer (CRC) remains the third most common cause of cancer death in the UK with a significant morbidity and mortality burden [1]. Incidence of CRC in patients in their second, third and fourth decade has steadily increased over the last 20 years [2]. Early onset colorectal cancer (EOCRC) has variable definitions, including CRC diagnosed before screening age, under 40 or 50 years. Regardless of definition, the rising incidence of EOCRC is well described across Western Europe, North America, and Australasia [2, 3]. The underlying aetiology for this trend remains unclear, but is likely multifactorial and includes obesity, hyperlipidaemia, excess alcohol consumption and sugar-sweetened beverages, among others [4, 5].

EOCRC exhibits distinct characteristics compared to later-onset CRC (LOCRC). Younger patients present with a greater proportion of left-sided tumours, more advanced stage disease at presentation and adverse tumour features, specifically mucinous or signet cell subtypes, and poor differentiation [6–8]. Concern that these factors bestow worse oncological outcomes on EOCRC patients, studies and reviews of how CRC outcomes differ between early- and late-onset disease have been performed and have reported conflicting results. Some studies report poor outcomes at all disease stages, while others report that EOCRC has favourable survival compared to LOCRC. Previous reviews and meta-analyses have focussed on examining whether EOCRC is a distinct entity from LOCRC by directly comparing younger and older cohorts using statistical adjustment for factors such as stage [3, 9–12].

Therefore, the aim of this study was to perform a systematic review of the literature comparing the overall survival of early- and late-onset CRC with stratification by TNM stage to control for the confounding effect of stage at presentation that has limited previous reviews. It also aims to explore other factors that may affect OS outcomes in EOCRC and LOCRC groups.

## Methods

This systematic review was conducted and reported in accordance with the PRISMA guidelines for systematic reviews[13]. The review was registered on the PROSPERO international prospective register of Systematic Reviews on 20 October 2024 (ID CRD42024563472) [14].

### Searches

The EMBASE, Ovid MEDLINE and Cochrane Library were searched using approved search terms across three concepts: colorectal cancer, survival outcomes, and early onset disease. Keywords and MeSH terms within each concept were separated by the Boolean operator ‘OR’. No filters were applied. The searches were current as of 30 August 2024. The search strategy was approved by a systematic review specialist librarian (JH) and is included in Appendix 1.

### Abstract screening

Search results were uploaded to Rayyan systematic review application, where duplicate records were removed. Abstracts were independently screened in duplicate by reviewers (NA, HL, FS, HB and UT) using the Rayyan systematic review application. No automation tools were used. The first round of screening identified papers reporting survival outcomes for non-metastatic CRC according to age group. Disagreements between reviewers were resolved by automatic inclusion of the title and abstract for full-text review. Inclusion criteria are outlined in Table 1.

**Table 1:** Inclusion and exclusion criteria.

Inclusion	Exclusion
Compared younger and older cohorts (age cut-off(s) specified)	Study reports outcomes for Stage IV CRC cancer only
Direct comparison of 5-year overall survival for individual disease stage(s)	No stratification by TNM stages I-III
Published after year 2000	Inclusion of patients under 50 years without older comparator cohort
Full text available in English	Systematic review or meta-analysis
	Case reports
	Series <10 patients
	Unpublished abstracts/conference proceedings

### Full paper review

Full manuscripts of all included studies were obtained through the North Bristol NHS Foundation Trust library service. Articles were reviewed and included if they reported a minimum of overall survival (OS) in EOCRC and LOCRC cohorts, stratified by TNM stage I-IV. EOCRC and LOCRC were defined using any age cut-off that separated patients under age 50 years. References of all included studies were manually screened for additional eligible studies. Where multiple studies utilised the same dataset, the most recent study with the largest patient cohort was selected. Data extraction was performed using a standardized proforma by three authors (NA, FS, HL).

### Primary outcome measure

The primary outcome measure was overall survival (OS) at five years, assessed using the log-rank test and estimated through Kaplan-Meier survival curves (KMC). Where available, absolute OS figures were used; otherwise, estimates were derived from KMC data. Studies were categorised according to whether they reported favourable survival for EOCRC, LOCRC, or no significant difference for each TNM stage. In cases where multiple age cohorts were reported, the cohort with the most favourable overall survival was recorded. Disease free survival (DFS) and cancer-specific survival (CSS) were not specified outcomes due to the heterogeneity in their definition across studies. Furthermore, some study cohorts included patients undergoing non-operative management and thus OS was considered a more meaningful outcome measure.

### Other outcome measures

Factors known to influence OS in addition to TNM stage, and incorporated into multivariate analyses of OS were also examined where reported. Data on study characteristics, including study period, population, geographic location, data source and survival outcome definitions, were collected (Table 2, UT, HB). No assumptions were made about missing information.

**Table 2:** Summary of included studies.

Study	Year	MINORS	Inclusion criteria	Incl	Exclusion criteria	Excl	Data source	OS Definition	Median Follow up (Months)	% lost to follow-up
Zhao [36]	2017	16	Age 18-74 Stage I-III Single primary tumour Confirmed adenocarcinoma ASA I-III	995	Emergency surgery Palliative surgery Neoadjuvant therapy Hereditary cancer	357	SCR	Surgery to death	50	11.8
Vasuvedan [16]	2020	14	All ages All CRC patients	902	Missing data	38	SCR	Diagnosis to death	19	32
Nakayama [17]	2020	15	All ages Stage II-III Confirmed adenocarcinoma Curative surgery	7724	Missing age data Lost to follow up	13518	Registry JSGCRC	Not specified	72 months	Not specified
Franklyn [19]	2021	18	Age >18 All CRC patients	167501	Neuroendocrine tumours Other malignancy	Not stated	Registry 8 UK Regional registries	Not specified	51.6	Not specified
Frostberg [20]	2022	20	Age 18-40, 66-75 All CRC patients	15191	Missing stage data	900	Registry DCCG	Diagnosis to death	65-83	0.19%
Goldvasar [21]	2016	12	Age 18-40, 51-92 All CRC patients	330	Not stated	Not stated	SCR	Diagnosis to death	65.9	Not specified
Yang [22]	2024	13	Age <40 or >70 All stages Confirmed adenocarcinoma	1599	Hereditary cancer IBD Family history of CRC Emergency surgery Palliative surgery Meets Amsterdam criteria	137	MCR	Surgery to death	44.2	Not specified
Murata [23]	2016	13	All ages Stage I-III Curative surgery Single primary tumour	2338	Hereditary cancer R1	Not stated	SCR	Surgery to death	43.2	Not specified
Saluja [24]	2014	15	All ages All CRC patients	166	IBD Hereditary cancer	6	SCR	Not specified	Not specified	Not specified
Abe [26]	2023		All Ages Curative surgery for primary CRC	980	Palliative surgery Incomplete records	542	SCR	Not specified	47 Months	Not specified
Son [27]	2023	18	All ages Stage I-III Confirmed adenocarcinoma Surgery with curative intent	1992	Stage IV Hereditary cancer Incomplete data	510	MCR	Surgery to death	Not specified	Not specified
Fontana [29]	2022	16	Included in IDEA database Stage II-III	14518	No SACT Some regimens	1831	Trial database IDEA	Not specified	69-72	Not specified
Okamoto [30]	2024	18	Ages >18 and <70 Stage I-III Confirmed adenocarcinoma Curative surgery Adjuvant chemotherapy	3324	IBD Hereditary cancer Synchronous CRC Other malignancy Missing data	12710	Registry JSGCRC	Not specified	Not specified	Not specified
Jin [31]	2021	14	Included in ACCENT database	35713	Data from studies with <50 patients in either age group Studies before 1990 Missing outcome data	Not stated	Trial database ACCENT	Randomisation to death	84	Not specified
Gao [35]	2022	19	All ages All CRC patients	34067	Other malignancy Recurrent CRC	1679	SCR	Not specified	Not specified	Not specified
Manjelievskaia [34]	2024	16	Aged 18-75 All CRC	3143	Missing age data Synchronous tumours Other malignancy	Not stated	Registry DODCR	Not specified	39.6	Not specified
Aguiar [28]	2020	13	Age >18 All CRC patients	2279	Not stated	Not stated	SCR	Diagnosis to death	Not specified	Not specified
Steele [28]	2014	17	Age >18 All CRC patients	6812	Stage 0 disease Hereditary cancer Other malignancy Recurrent cancer Improperly coded mortality data	1141	Registry ACTUR	Not specified	56.4	Not specified
O’Sullivan [25]	2022	18	Age >18 Confirmed adenocarcinoma	8748	Carcinoid tumour Appendix tumour	Not stated	Registry ACR	Not specified	Not specified	Not specified
Kolarich [33]	2018	18	Age 20-75 Stage I-III Rectal cancer Curative surgery	43106	Stage 0 or IV Missing data R1 Unable to have chemotherapy due to comorbidity or death	200560	Registry NCDB	Not specified	Not specified	Not specified
Liu [32]	2024	16	All ages Stage III Rectal cancer Neoadjuvant therapy followed by TME	757	Off guideline neoadjuvant therapy Delay >6 months from radiotherapy to surgery	42	SCR	Diagnosis to death	Not specified	Not specified
Poles [39]	2016	18	All ages All CRC patients Confirmed adenocarcinoma	1462150	Other malignancy Missing data Other histological subtypes Lost to follow up	Not stated	Registry NCDB	Not specified	Not specified	Not specified

*SCR* Single centre retrospective, *MCR* Multi centre retrospective, *SACT* Systemic anti-cancer treatment, *ACTUR* Department of Defence Automated Central Tumour Registry, *NCDB* National Cancer Database, *ACR*: Alberta Cancer Registry, *JSGCRC* The Japanese Study Group for Follow-Up of Colorectal Cancer, *ACCENT* Adjuvant Colon Cancer ENdpoint database, *DCCG* Danish Colorectal Cancer Group data base and in the Danish Cancer Registry, *IDEA* The International Duration Evaluation of Adjuvant Chemotherapy collaboration.

### Methodological assessment

The methodological quality of the included studies was assessed using the Methodological Index for Non-randomized Studies (MINORS) criteria, with scoring conducted independently by two authors (HB, UE). Disagreements were reviewed and settled by the third author (NA) (Table 2) [15].

### Ethics approval

Ethical approval was not required for this study as it was a systematic review of previously published studies and did not involve primary data collection from human participants.

## Results

**Fig. 1:**
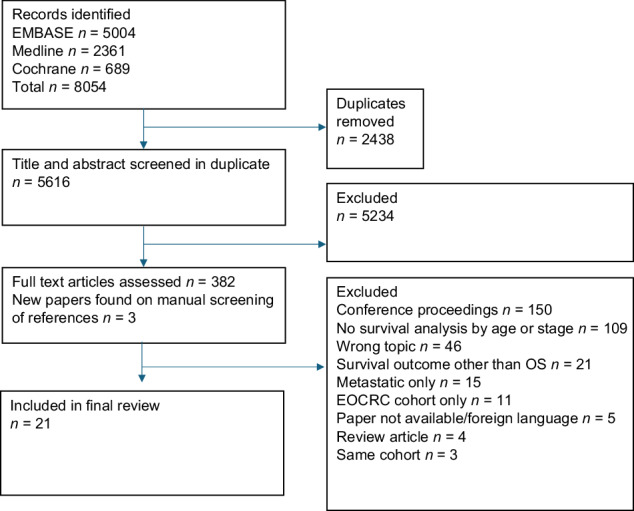
PRIMSA flowchart.

A total of 8054 articles were identified using the search strategy defined above. After duplicate exclusion, 5616 titles and abstracts were screened. After screening, 382 full-texts were assessed, and 24 studies met the inclusion criteria that described stage-stratified survival outcomes in 29,199 patients with EOCRC and 303252 patients with LOCRC [16–37]. Three papers were excluded due to overlapping patient cohorts [38–40]. One excluded study is discussed separately as it is the only paper to include a paediatric subgroup [39]. Prisma flowchart is shown in Fig. 1.

### Inclusion criteria, survival definition and risk of bias

Inclusion criteria and risk of bias varied widely across studies (Table 2). There was heterogeneity between studies surrounding the definition of OS, which is summarised in Table 2. Three studies reported separate outcomes for colon and rectal cancer [17, 20, 28]. Two studies reported outcomes for rectal cancer alone [32, 33], while two other studies reported outcomes from adjuvant chemotherapy trials in colon cancer [29, 37]. The MINORS varied across included studies and ranged from 14 to 23. Most studies were subject to selection bias secondary to their pre-specified inclusion and exclusion criteria. Studies reported variable patient loss to follow-up and excluded patients based on criteria. No studies performed a sample size calculation.

### Definition of EOCRC and overall survival according to age group and stage

**Fig. 2:**
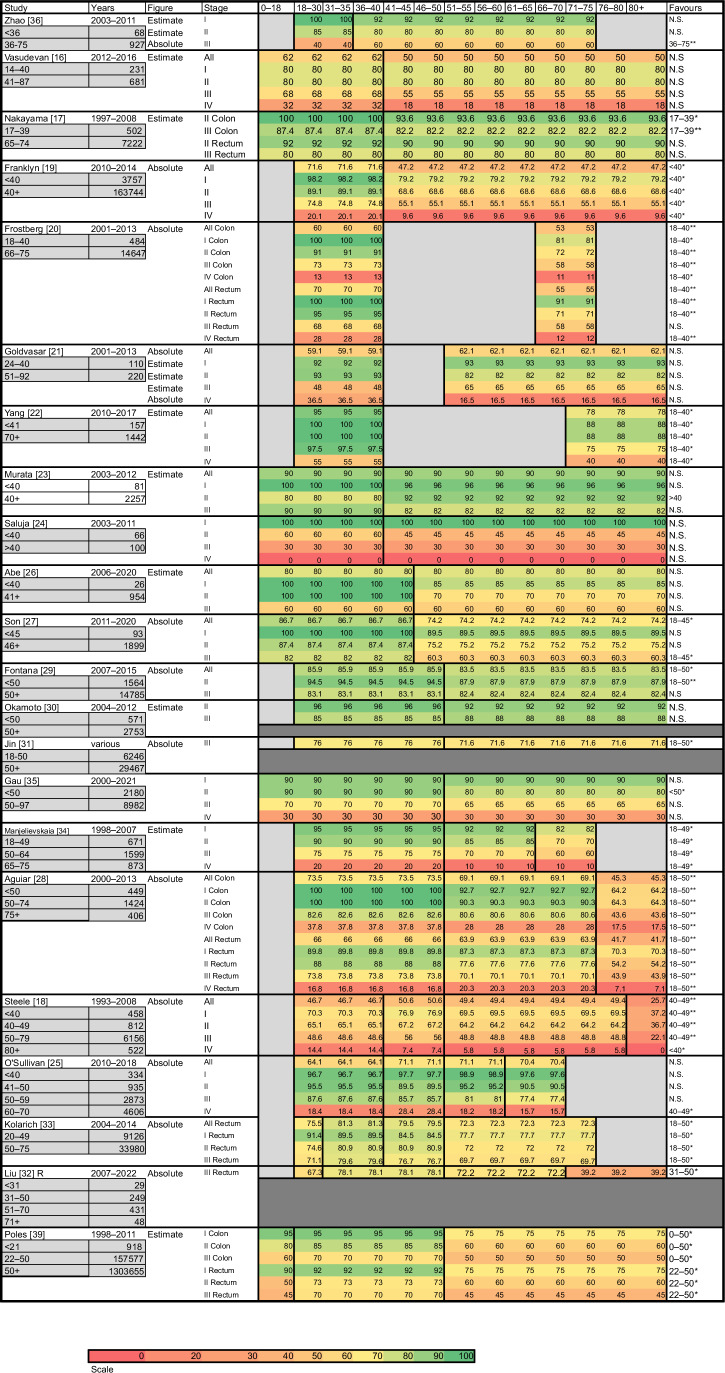
Summary of papers including age categories used, study years, sample size, how data was extracted, disease stages reported and statistical significance of findings (Not significant—N.S. *p* > 0.05, *p < 0.01, **p < 0.001). Study identified by first author name and reference number. Age categorizations and number in each group. 5-year overall survival, either absolute (exact figures published in a table) or estimated from Kaplan–Meier curve. Colours continuous gradient from 0 to 100 (see below).

The age cut-off for defining EOCRC, inclusion and exclusion criteria and definition of OS varied widely among studies, influencing how survival outcomes were reported and thus precluding any meaningful meta-analysis. Only studies reporting 5 years OS according to age and stage were included (Fig. 2).

### Age 35 years

Zhao et al included 68 patients (6.8%) under 35 years and 927 patients (93.2%) over 35 years [37]. Stage III CRC patients aged under 35 years had significantly worse OS when compared to patients aged between 36-75 years.

### Age 40 years

Five studies compared patients under 40 years to all patients over 40 years with no upper age limit [16, 17, 19, 23, 24]. Three other studies compared patients under 40 years to late-onset CRC cohorts of varying age ranges [20–22]. There was a combined total of 5388 patients (2.8%) aged under 40 years and 189,632 aged over 40 years (97.2%) in these studies. Studies reported mixed findings: three studies found no difference in OS between EOCRC and LOCRC for all TNM stages [16, 21, 24], Murata et al showed favourable survival in patients over 40 years old with stage II disease [23]. Yang et al and Franklyn et al demonstrated favourable OS for EOCRC across all TNM stages [19, 22]. Nakayama et al. and Frostberg et al reported improved survival in EOCRC for all stages of colonic cancer but no survival difference in stage III rectal cancer[17, 20].

### Age 45 years

Two studies used a cut-off of 45 years that included a total of 119 (4.0%) patients under 45 years and 2853 over 45 years (96.0%) [27, 41]. Son et al. showed favourable survival for EOCRC with stage III disease [27].

### Age 50 years

Ten studies used a cut-off of 50 years that included a total of 23624 EOCRC patients (17.7%) and 109840 LOCRC patients (82.3%) [19, 26, 29–31, 33–36, 38]. Five studies used only two age groups [29, 30, 34, 35, 37]. Okamoto et al was the only study to find no difference in survival for any stage [30]. Of the remaining four studies that compared only two age groups, favourable survival for the EOCRC cohort was reported for at least one TNM disease stage [29, 34, 35, 37]. Five other studies reported outcomes between age groups within the EOCRC or LOCRC cohorts [18, 25, 28, 32, 33]. O’Sullivan et al demonstrated favourable survival for stage IV disease for 41–50-year-olds when compared to patients in groups that were both younger and older, but no survival difference in patients with non-metastatic disease [25]. Aguiar et al found patients under 50 to have the best OS at all stages compared to all age sub-groups within the LOCRC cohort [28]. Steele and Liu demonstrated improved survival in patients aged under 50 years, with patients aged 40-50 years having better OS when compared to groups aged <40 years and <30 years. Kolarich et al did not perform a statistical comparison between the youngest age groups but noted a trend towards worse OS in the 18-30 years age group, compared to 30-40 and 40-50 [33].

### Outcomes of elderly age groups reported separately

Three studies reported outcomes of elderly patients in their 7th and 8th decades separately. In all cases, the very elderly patients had significantly worse OS than all other younger age-groups [18, 28, 32]. One study reported outcomes of 65–75-year-olds separately, and these were significantly worse than for all younger age groups [34].

### Paediatric age groups

Poles et al. reported outcomes of 918 paediatric patients under 21 years, but this was not included in the main review as data overlapped data with the study by Kolarich et al. [33, 39]. Of note, patients under 21 years displayed worse survival compared to older age groups within the EOCRC cohort for stage II and III disease.

### Rectal cancer

Four studies reported outcomes for colon and rectal cancer separately. All these studies demonstrated better OS from colon cancer compared to rectal cancer [17, 20, 28, 39]. Okamoto et al. showed that male patients aged under 50 years with stage III rectal cancer had worse survival compared to patients in all other age groups in the late-onset cohort [40].

### Multivariate analysis of overall survival

Ten of the included studies performed a multivariate analysis of factors influencing OS [20–23, 27, 32, 33, 35–37]. Four studies performed multivariate analyses for subgroups, including colon vs rectal cancer [20], whether results of molecular markers were available (MMR, BRAF, KRAS) [37], and EOCRC vs LOCRC [22, 33]. Multiple factors were found to influence overall survival. In seven studies, age at onset remained a prognostic factor[20, 23, 27, 32, 33, 36, 37]. Other factors influencing OS included age, gender, tumour stage, subtype, grade and differentiation and presence of lymphovascular invasion, among others.

## Discussion

**Fig. 3:**
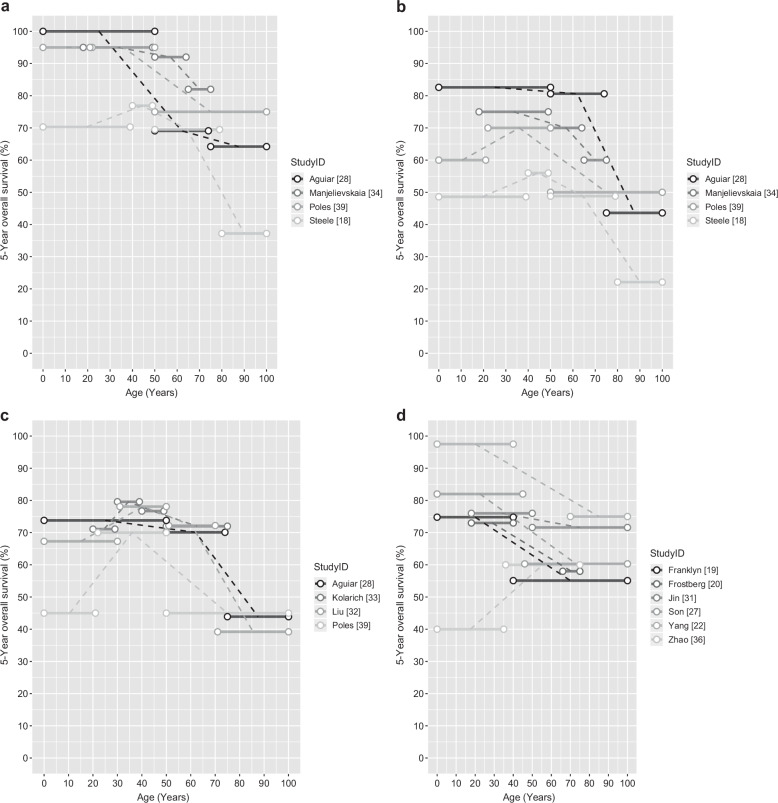
Studies reporting statistically significant differences in 5-year overall survival according to age group, stratified by TNM stage. **a**–**c** show studies that compared more than two age categories. **d** shows studies that compared only two specified age categories. Results are expressed as percentages of 5-year overall survival. **a** Stage I colorectal cancer, **b** Stage III colorectal cancer, **c** Stage III rectal cancer, **d** Stage III colorectal cancer.

This systematic review comparing survival outcomes between patients with early- and late-onset is unique in that it has attempted to account for the confounding effect of stage at presentation. EOCRC is typically more advanced at presentation, and when comparing all stages, this could be responsible for perceived worse OS compared to LOCRC [12]. The review highlights the multiple definitions used by studies in reporting EOCRC and has attempted to account for this in the description of the results. Such variability has precluded meta-analysis from being conducted. Despite this, clear themes have emerged. Survival across all TNM stages is better in the EOCRC cohorts, however, in studies that have sub-divided their EOCRC cohort into multiple age groups, survival among the very youngest (cut-offs less than 40 years) appears worse when compared to older age groups (Fig. 3). This was true for rectal cancer where young male patients had the worst survival outcomes, although both studies had fewer than 100 patients in their EOCRC cohorts, and used age cut-offs of 35 and 40, respectively [23, 36]. The remaining studies demonstrated either equivalent or favourable stagewise OS in EOCRC.

At first glance, these findings present a compelling argument for improved survival in EOCRC, but some indications point towards worse outcomes in certain subgroups. In four studies, the youngest EOCRC subgroup had significantly poorer OS compared to older EOCRC patients [18, 25, 32, 33] (Fig. 3). Younger subgroups also conferred less survival benefit in more advanced stage and in rectal cancer than in colon cancer in three studies. In two studies, this was noted to be more pronounced among male patients [17, 20, 39, 40]. In addition, several other factors influencing overall survival beyond age or disease stage were identified.

It is becoming widely recognised that EOCRC patients tend to present at a later stage and are more likely to have left-sided or rectal tumours with aggressive histological including poor differentiation, signet cell or mucinous subtypes. Tumours are also more likely to exhibit MSI high or BRAF mutations [3, 9–12]. A large, recent meta-analysis conducted by Carbone et al compared multiple survival outcomes in patients under and over 50. The study did not demonstrate that poor oncologic features translate into worse comparative OS, disease-free survival (DFS) or cancer-specific survival (CSS), despite attempting to control for variation in case mix (excluding hereditary CRC). After excluding influential studies, DFS was shown to be worse in EOCRC patients, especially young rectal cancer patients [12]. While these studies are detailed meta-analyses performed to a high standard, the heterogenous nature of existing literature means that nuance and detail are lost. For example, despite death being a robust and consistent endpoint, challenges still exist when comparing OS between different age groups, making such comparisons less valid regardless of sample size. Studies using Surveillance, Epidemiology and End Results (SEER) database by Wang and Chen demonstrate that the cause of death in the first five years after diagnosis of CRC varies significantly in those aged under 50 and over 65. CRC accounted for over 85% of deaths in EOCRC patients under 50 but only 70% in those over 65. This difference is even more pronounced in early-stage disease, with less than 30% of deaths in stage I CRC patients aged 65–75 years old [42, 43]. These findings suggest that higher non-cancer-related mortality in older patients reduces the observed OS, something which is not happening in EOCRC cohorts, where overall survival is a true cancer-related metric. This may explain why some studies reported worse outcomes in younger EOCRC subgroups [18, 32, 33]. This difference is accentuated in studies which included all patients with CRC, not just those treated surgically, as elderly patients are more likely to be treated without surgery, and such patients have been have shown to have extremely poor prognosis [18, 19, 34, 44].

Multiple factors beyond disease stage also vary between CRC age groups and influence prognosis. Hereditary and IBD-related cancers have distinct survival patterns, and are over-represented in EOCRC, especially the youngest age groups [45]. On the other hand, LOCRC patients are more likely to have screen-detected cancers. Evidence suggests that screening programs for EOCRC result in favourable OS [46].

### Strength and limitations

Previous studies have applied statistical methods to strengthen their conclusions. In our systematic review, we took a detailed approach to understand factors influencing overall survival. By examining the determinants of survival in depth, we highlighted limitations of simply comparing outcomes between older and younger patients. Our findings reinforce that EOCRC is a distinct clinical entity that remains incompletely understood. Furthermore, relying solely on age cutoffs does not provide us with sufficient insight.

A limitation of our study includes the focus on OS and not using CSS or DFS as an outcome, as using CSS and DFS metrics would tell us more about tumour behaviour. However, few of the included studies specified the proportion of patients that had resected disease, thus limiting the usefulness of these survival metrics.

## Conclusions

While asking “does EOCRC have worse prognosis than LOCRC?” appears straightforward, the answer is complex. There is more to answering this question than merely comparing younger and older age groups and their survival outcomes. The increasing incidence of EOCRC, along with its aggressive phenotype and late-stage presentation, is concerning for clinicians, patients, and society. Prognosis varies significantly across EOCRC and LOCRC cohorts, and direct comparisons using age cut-offs fail to capture the full picture.

Rather than focusing on survival comparisons between younger and older cohorts, future research should centre on developing large, prospective datasets that are highly granular with detailed data on age, stage, disease sub-type, presence of hereditary cancer or inflammatory bowel disease, histological features and treatment received. This will provide a more accurate picture of prognosis and thus guide treatment strategies. Above all else, survival from early stage EOCRC is very favourable, which highlights the importance of developing novel screening programmes for early detection.

## Supplementary information

Appendix 1

## Data Availability

All data related to this manuscript is kept by the corresponding author and is available on request
