# Supplementary material for: A comparison of overall survival from early-onset and late-onset colorectal cancer stratified by TNM stage: a systematic review
Source: Br J Cancer. 2026 May 4;135(3):461–9. doi: 10.1038/s41416-026-03426-w (PMC13372811; doi:10.1038/s41416-026-03426-w)
Supplement: Supplementary file 1 — Appendix 1 [file 41416_2026_3426_MOESM1_ESM.docx]

# **Appendix 1**

# Search Strategy Documentation

45-093 30_Aug_2024_Survival outcomes from non-metastatic early onset colorectal cancer

**Search Methodology**

The search strategy was created in collaboration with a medical librarian. We searched Medline (via OVID), EMBASE (via OVID) and Cochrane from inception to 30/8/2024. The search included keyword and subject terms relating to “Colorectal Neoplasms”, “early-onset” and “follow up/ survival”, and search filters were used to limit to “2000”, “Humans”.

To be amended added to final document by main author(s)

**Search Summary**

| **Database Name** | **Platform** | **Date Coverage** | **Date of Search** | **# of results** |
| --- | --- | --- | --- | --- |
| 1. Medline | OVID | 2000 to August 29, 2024 | 30/8/2024 | 2361 |
| 2. EMBASE | OVID | 2000 to August 29, 2024 | 30/8/2024 | 5004 |
| 3.Cochrane |  | inception to August 29, 2024 | 30/8/2024 | 775 |
|  |  |  | **TOTAL** | 8,140 |

**Search Strategies**

**1. Medline (OVID)**

**Date of Search: 30/8/2024**

1 exp Colorectal Neoplasms/ 248731

2 exp Colonic Neoplasms/ 84796

3 exp Rectal Neoplasms/ 56324

4 ((colorectal* or colon* or bowel* or rectal or rectum or sigmoid) adj5 (cancer or neoplasm* or tumor* or tumour or carcinom* or adenocarcinom* or adenom* or lesion*)).tw,kf. 310973

5 1 or 2 or 3 or 4 365396

6 (early adj3 onset).tw,kf. 58188

7 early-onset.tw,kf. 50256

8 (young* adj3 onset).tw,kf. 6511

9 young-onset.tw,kf. 2147

10 (young* adj4 (people or person or adult* or patient*)).tw,kf. 320193

11 6 or 7 or 8 or 9 or 10 379445

12 prognosis/ 619079

13 treatment outcome/ 1205415

14 survival analysis/ 146446

15 Follow-Up Studies/ 701945

16 exp Death/ 169140

17 exp Mortality/ 431418

18 (death or die or dying).tw,kf. 1028898

19 prognos*.tw,kf. 879584

20 mortality.tw,kf. 1065940

21 surviv*.tw,kf. 1521052

22 (disease adj3 (outcome* or progess* or course or recurr* or predict*)).tw,kf. 185098

23 (recovery adj3 (outcome* or progess* or expectation* or course or predict*)).tw,kf. 15173

24 "natural history".tw,kf. 57543

25 12 or 13 or 14 or 15 or 16 or 17 or 18 or 19 or 20 or 21 or 22 or 23 or 24 5246414

26 5 and 11 and 25 2870

27 exp ANIMALS/ not HUMANS/ 5253024

28 26 not 27 2855

29 limit 29 to (english language and yr="2000 -Current") 2361

**2. EMBASE (OVID)**

**Date of Search: 30/8/2024**

1 exp colorectal tumor/ 487667

2 exp colon tumor/ 178122

3 exp rectum tumor/ 81448

4 ((colorectal* or colon* or bowel* or rectal or rectum or sigmoid) adj5 (cancer or neoplasm* or tumor* or tumour or carcinom* or adenocarcinom* or adenom* or lesion*)).tw,kf. 451827

5 1 or 2 or 3 or 4 581638

6 (early adj3 onset).tw,kf. 86347

7 early-onset.tw,kf. 74509

8 (young* adj3 onset).tw,kf. 10675

9 young-onset.tw,kf. 3775

10 (young* adj4 (people or person or adult* or patient*)).tw,kf. 454565

11 6 or 7 or 8 or 9 or 10 542397

12 prognosis/ or cancer prognosis/ 966063

13 treatment outcome/ 1013039

14 survival analysis/ 58074

15 follow up/ 2239979

16 exp death/ 2141187

17 exp mortality/ 1469461

18 (death or die or dying).tw,kf. 1438744

19 prognos*.tw,kf. 1322154

20 mortality.tw,kf. 1580086

21 surviv*.tw,kf. 2234491

22 (disease adj3 (outcome* or progess* or course or recurr* or predict*)).tw,kf. 295264

23 (recovery adj3 (outcome* or progess* or expectation* or course or predict*)).tw,kf. 21601

24 "natural history".tw,kf. 82137

25 12 or 13 or 14 or 15 or 16 or 17 or 18 or 19 or 20 or 21 or 22 or 23 or 24 8005462

26 5 and 11 and 25 5625

27 exp animal/ not human/ 5453001

28 26 not 27 5579

29 limit 28 to english language 5298

30 limit 29 to (english language and yr="2000 -Current") 5004

31 exp colorectal tumor/ 487667

32 exp colon tumor/ 178122

33 exp rectum tumor/ 81448

34 ((colorectal* or colon* or bowel* or rectal or rectum or sigmoid) adj5 (cancer or neoplasm* or tumor* or tumour or carcinom* or adenocarcinom* or adenom* or lesion*)).tw,kf. 451827

35 31 or 32 or 33 or 34 581638

36 (early adj3 onset).tw,kf. 86347

37 early-onset.tw,kf. 74509

38 (young* adj3 onset).tw,kf. 10675

39 young-onset.tw,kf. 3775

40 (young* adj4 (people or person or adult* or patient*)).tw,kf. 454565

41 36 or 37 or 38 or 39 or 40 542397

42 prognosis/ or cancer prognosis/ 966063

43 treatment outcome/ 1013039

44 survival analysis/ 58074

45 follow up/ 2239979

46 exp death/ 2141187

47 exp mortality/ 1469461

48 (death or die or dying).tw,kf. 1438744

49 prognos*.tw,kf. 1322154

50 mortality.tw,kf. 1580086

51 surviv*.tw,kf. 2234491

52 (disease adj3 (outcome* or progess* or course or recurr* or predict*)).tw,kf. 295264

53 (recovery adj3 (outcome* or progess* or expectation* or course or predict*)).tw,kf. 21601

54 "natural history".tw,kf. 82137

55 42 or 43 or 44 or 45 or 46 or 47 or 48 or 49 or 50 or 51 or 52 or 53 or 54 8005462

56 35 and 41 and 55 5625

57 exp animal/ not human/ 5453001

58 56 not 57 5579

59 limit 59 to (english language and yr="2000 -Current") 5004

**3. Cochrane**

**Date of Search: 30/8/2024**

#1 MeSH descriptor: [Colorectal Neoplasms] explode all trees 12790

#2 MeSH descriptor: [Colonic Neoplasms] explode all trees 2567

#3 MeSH descriptor: [Rectal Neoplasms] explode all trees 2889

#4 ((colorectal* or colon* or bowel* or rectal or rectum or sigmoid) NEAR/5 (cancer or neoplasm* or tumor* or tumour or carcinom* or adenocarcinom* or adenom* or lesion*)) 30979

#5 #1 or #2 or #3 or #4 31201

#6 (early NEAR/3 onset) 3371

#7 early-onset 2721

#8 young* NEAR/3 onset 276

#9 young-onset 78

#10 (young* NEAR/4 (people or person or adult* or patient*)) 133057

#11 #6 or #7 or# 8 or #9 or #10 136054

#12 MeSH descriptor: [Prognosis] this term only 21089

#13 MeSH descriptor: [Treatment Outcome] this term only 193527

#14 MeSH descriptor: [Survival Analysis] this term only 11168

#15 MeSH descriptor: [Follow-Up Studies] this term only 75012

#16 MeSH descriptor: [Death] explode all trees 3554

#17 MeSH descriptor: [Mortality] explode all trees 18923

#18 (death or die or dying) 98519

#19 prognos* 61154

#20 mortality 127827

#21 surviv* 157199

#22 (disease NEAR/3 (outcome* or progess* or course or recurr* or predict*)) 50313

#23 (recovery NEAR/3 (outcome* or progess* or expectation* or course or predict*)) 4419

#24 "natural history" 3055

#25 #12 or #13 or #14 or #15 or #16 or #17 or #18 or #19 or #20 or #21 or #22 or #23 or #24 526100

#26 #5 and #11 and #25 775

#27 MeSH descriptor: [Animals] explode all trees 898169

#28 MeSH descriptor: [Humans] this term only 894506

#29 #27 NOT #28 3663

#30 #26 NOT #29 775
